# *TRPM2* SNP genotype previously associated with susceptibility to *Rhodococcus equi* pneumonia in Quarter Horse foals displays differential gene expression identified using RNA-Seq

**DOI:** 10.1186/s12864-016-3345-3

**Published:** 2016-12-05

**Authors:** Cole M. McQueen, Canaan M. Whitfield-Cargile, Kranti Konganti, Glenn P. Blodgett, Scott V. Dindot, Noah D. Cohen

**Affiliations:** 1 Department of Large Animal Clinical Sciences, Texas A&M University College of Veterinary Medicine & Biomedical Sciences, College Station, TX USA; 2 Texas A&M Institute for Genome Sciences and Society, Texas A&M University, College Station, TX USA; 3 6666 Ranch, 1102 Dash For Cash Road, Guthrie, TX USA; 4 Department of Veterinary Pathobiology, Texas A&M University College of Veterinary Medicine & Biomedical Sciences, College Station, TX USA

**Keywords:** *Rhodococcus equi*, Horses, RNA-Seq, Gene expression, Pneumonia

## Abstract

**Background:**

*Rhodococcus equi* (*R. equi*) is an intracellular bacterium that affects young foals and immuno-compromised individuals causing severe pneumonia. Currently, the genetic mechanisms that confer susceptibility and/or resistance to *R. equi* are not fully understood. Previously, using a SNP-based genome-wide association study, we identified a region on equine chromosome 26 associated with culture-confirmed clinical pneumonia. To better characterize this region and understand the function of the SNP located within *TRPM2* that was associated with *R. equi* pneumonia, we performed RNA-Seq on 12 horses representing the 3 genotypic forms of this SNP.

**Results:**

We identified differentially expressed genes in the innate immune response pathway when comparing homozygous A allele horses with the AB and BB horses. Isoform analyses of the RNA-Seq data predicted the existence of multiple transcripts and provided evidence of differential expression at the *TRPM2* locus. This finding is consistent with previously demonstrated work in human cell lines in which isoform-specific expression of *TRPM2* was critical for cell viability.

**Conclusions:**

This work demonstrates that SNPs in *TRPM2* are associated with differences in gene expression, suggesting that modulation of expression of this innate immune gene contributes to susceptibility to *R. equi* pneumonia.

## Background

*Rhodococcus equi* (*R. equi*) is a pathogen that predominantly affects young foals causing pneumonia as well as extra-pulmonary disorders [1–5]. Currently, there is no approved vaccine for protection against *R. equi* pneumonia, and other preventative interventions, such as transfusion of hyperimmune plasma, are expensive, labor-intensive, and incompletely effective [4, 6]. Isolates of *R. equi* that cause disease in foals must bear the virulence associated protein A (VapA) gene in a plasmid; however, presence of the plasmid and VapA expression alone is not sufficient to cause disease, indicating that host factors are of great importance [7, 8]. In addition to anecdotal reports of some mares having multiple infected foals while other mares in the same environment never have an affected foal, several candidate gene studies suggest a genetic basis for *R. equi* susceptibility [9–13]. Because of gene-selection bias and phenotypic misclassification, the associations from candidate gene studies have been weak and potentially biased [14].

Recently, our laboratory identified a region on chromosome 26 associated with *R. equi* pneumonia in a genome-wide association study (GWAS) [15] using a commercially available single nucleotide polymorphism (SNP) array [15, 16]. Four SNPs were associated with clinical disease in a region spanning several predicted genes. One of the SNPs was well suited to serve as a marker because it was located within a candidate gene (*viz.*, the transient receptor potential cation channel, subfamily M, member2 [*TRMP2*]) and could be easily and accurately genotyped [15]. This SNP was associated with 3 genotypes designated AA, AB, and BB alleles. Because SNPs are merely indicators of location and are very rarely actual causal mutations, it remains unclear which genes in this region might explain the observed association of genotype with disease. More importantly, these SNPs alone do not provide any functional information regarding the relationship between genotype and phenotype across this region. The marker SNP in *TRPM2* is a synonymous substitution and does not change the amino acid or protein sequence and is thus unlikely to be causally associated with disease. It is biologically plausible, however, that this SNP is a marker for other genetic elements nearby that might regulate gene expression. Thus, other approaches including investigating gene expression are necessary to further understand the observed association of the *TRPM2* SNP and *R. equi* pneumonia.

Investigating the whole transcriptome using RNA-Sequencing (RNA-Seq) provides an unbiased approach for gene expression analysis, including the analysis of alternatively spliced transcripts (*viz*., isoforms), [17] yielding greater possibility for identifying true expression phenotypes associated with the genotype(s) of interest. RNA-Seq can also be used to identify and examine unnanotated genes and isoforms. The principal aims of this study were: 1) to determine whether gene expression (including analysis of isoforms) in the *TRPM2* gene region was associated with the marker genotypes for the *TRPM2* SNP (*i.e.*, AA, AB, or BB alleles), using RNA-Seq (targeted approach); and, 2) to perform associations of gene expression across the transcriptome with the TRPM2 genotypes (untargeted approach). As a secondary aim, we examined the association of gene expression across the transcriptome with history of *R. equi* pneumonia. The objectives of this study were to identify whether the marker genotype identified in *TRPM2* was an indicator of differential expression, and to identify any other genes that were differentially expressed between the *TRPM2* SNP genotypes. Completing these objectives would allow us to find key biological pathways and processes involved in susceptibility to *R. equi* pneumonia [18–20].

## Methods

### Study population

The study population was derived from the source population for our GWAS described above (*viz.*, foals born during 2011 at the 6666 Ranch) [15], which had a cumulative incidence of *R. equi* pneumonia of 17% during 2011. Briefly, foals were monitored for clinical signs of fever, cough, nasal discharge, and lethargy which were suggestive of pneumonia. Foals with clinical signs of pneumonia were examined using thoracic ultrasonography, and foals with areas of pulmonary abscessation or consolidation were subjected to tracheobronchial aspiration for cytologic examination and microbiologic culture of aspirate fluid. A foal was considered to have clinical *R. equi* pneumonia when it had clinical signs of pneumonia, ultrasonographic evidence of pulmonary abscesses or consolidation, and a positive culture of *R. equi* from a tracheobronchial aspirate with cytological evidence of septic inflammation [15].

For the current study, 12 horses were randomly selected from 51 horses remaining at the 6666 Ranch that were part of the source population used in 2011. Using methods described previously, [15] *TRPM2* genotypes were then determined for these 12 horses (AA, *n* = 3; AB, *n* = 4; and BB *n* = 5). This sample size was determined by the funding available for this project and the cost for RNA-Seq.

### Sample collection and RNA-Seq

A whole blood sample (5 mL) was collected by jugular venipuncture into 2 Paxgene RNA Vacutainer tubes (PreAnalytiX, Hombrechtikon, Switzerland) at the 6666 Ranch, Guthrie, TX, June 25, 2014, to permit the RNA to be stabilized for transport to Texas A&M University. Total RNA was isolated using the MagMax Paxgene RNA purification kit (Life Technologies). RNA-Sequencing libraries were generated using the TrueSeq RNA preparation kit (Illumina) with a polyA selection step. The samples were pooled and sequenced (125-base-pair [bp], paired-end sequencing) on 2 lanes of a HiSeq 2500 (Illumina) to account for lane artifacts. The Texas A&M AgriLife Genomics and Bioinformatics core generated the RNA-Seq libraries and performed the RNA-Seq reactions. The raw data of the RNA-Seq reactions was processed using bioinformatic tools provided by the Texas A&M Institute for Genome Sciences and Society (TIGSS). FASTQ reads were de-multiplexed and assessed for quality using FastQC. Duplicate samples were merged and aligned to the equine reference genome assembly (EquCab2) using *Tophat (*version 2.0.14) [21].

### Gene expression analysis for genotypic and phenotypic comparisons

The primary aim of the study was to identify differentially expressed genes, using either a targeted (TRPM2 gene region, including isoforms) or untargeted (transcriptome-wide) approach, between horses with the *TRPM2* AA genotype (*n* = 3) and horses with either the *TRPM2* AB or BB genotypes (*n* = 9). The AB and BB *TRPM2* genotypes were combined on the basis of our previous results indicating that the odds of disease were similar in comparisons between horses with the AA *TRPM2* genotype relative to the AB genotype (OR = 4.3; *P* = 0.0017), the BB genotype (OR = 3.6; *P* = 0.0574), or combined genotypes (OR = 3.7; *P* = 0.0006) [15]. A secondary aim was to compare differential gene expression between horses that had developed *R. equi* pneumonia as foals (*n* = 2) and horses that had not developed clinical signs of pneumonia as foals (*i.e.*, subclinical pneumonia or no pneumonia; *n* = 10), independent of *TRPM2* genotype.

Sequencing reads were aligned to the equine genome using *Tophat* with the default parameters. *EdgeR*, which operates within the R statistical package (Version 3.0.1; R Statistical Project), and *cuffdiff* were used to identify differentially expressed genes using the Ensembl equine gene annotation [build 83] [21–26]. For the *edgeR* analysis, read counts of each gene were first determined using *HTSeq* [27] with the intersection nonempty parameter to account for ambiguous reads. The resulting count table was filtered to remove genes in which 0 reads mapped in all samples. The tabulated read counts were then normalized relative to library size and tag-wise and common dispersions estimated. Differentially expressed genes were defined as those genes having a false discovery rate (FDR) ≤ 0.05 that were identified with the *exactTest* function. For the *cuffdiff* analysis the default settings were used with the exception of the minimum isoform fraction setting (F - 0). Differentially expressed genes were defined as those having a q-value (FDR) ≤ 0.05.

Biological pathway analysis was performed using the Ingenuity Pathway Analysis (IPA) (Qiagen, Venlo, Netherlands; www.ingenuity.com) tool-kit. Output files from both *edgeR* and *cuffdiff* were used in their respective pathway analyses. Input files for IPA used the following information from the *edgeR* or *cuffdiff* outputs: a column containing an Ensembl gene identifier, a corresponding gene name where applicable, the log fold-change of expression for each gene between the 2 groups tested, and a FDR for each gene tested.

Visualization of RNA-Seq read coverage across the region of interest was carried out using *BEDTools* and the University of California Santa Cruz (UCSC) online genome browser using aligned reads from the previously described *Tophat* mapping step [28, 29]. The *cufflinks* function of the *Tophat* tool suite was used to identify novel transcripts at the *TRPM2* locus [21]. The Ensembl annotated *TRPM2* gene model was then replaced with the *TRPM2* assembled transcripts generated through *Cufflinks*. Analysis of the *TRPM2* isoforms was performed using *cuffdiff*.

The assembled *TRPM2* transcripts were verified by polymerase chain reaction (PCR). Briefly, equine RNA was isolated using the Ambion PureLink™ RNA isolation kit (Ambion, Waltham, MA) and cDNA synthesized using the iScript™ cDNA synthesis kit (BIO-RAD, Hercules, CA). PCR probes were developed using the Primer3 software [30] for the 3′ end of predicted *TRPM-1* with complementary reverse primers targeted at the 5′ and 3′ ends of *TRPM2-2*. Cycling conditions were as follows: 94 °C for 2 min, 94 °C for 30 s to 58 °C for 45 s to 72 °C for 1 min 30 times, followed by 72 °C for 3 min. PCR products were resolved on a 1% agarose gel.

## Results

### RNA-seq of horse samples

RNA-seq generated an average of 33.7 million paired-end reads per sample of which 78.7% (*viz.,* 26.5 million) uniquely aligned to the equine genome. With the exception of 1 sample (*viz.,* foal 45), all samples had base quality scores (Phred) > 30.

### Analysis of TRPM2 region gene expression and assembled transcripts (targeted approach)

**Fig. 1:**
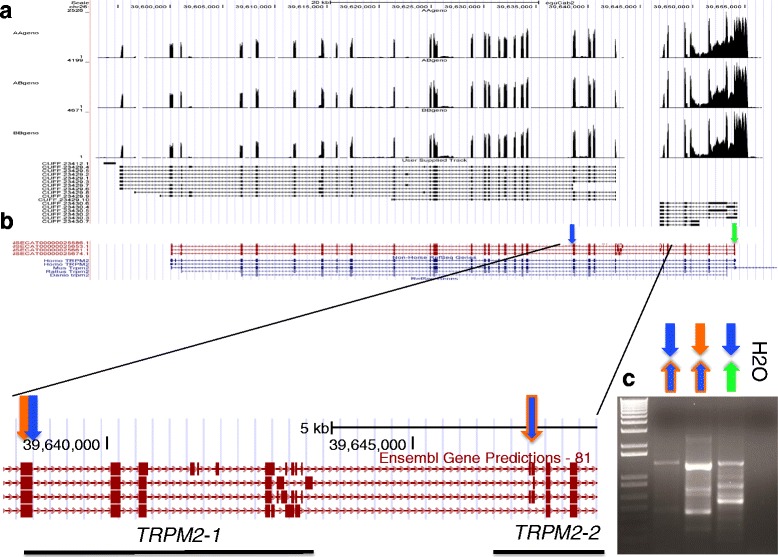
**a** Bedgraphs representing RNA-Seq coverage of *TRPM2* for each genotype from top to bottom: AA, AB, and BB. **b** *Cufflinks* output of *TRPM2* novel isoform overlaying the equine Ensembl predicted gene annotations. **c** PCR amplicons stained with EtBr reveal splicing from *cuffdiff* identified *TRPM2-1* to *TRPM2-2* as well as multiple isoforms. Primer locations indicated by colored arrows in panel **b** and **c** and resulting amplicons of primer pairs represented in in panel **c**

Neither the TRPM2 nor adjacent genes were differentially expressed between the AA and non-AA genotypes. Visualization of the aligned RNA-Seq reads relative to the *TRPM2* gene annotation (Ensembl equine 83 and Non-horse RefSeq annotation) suggested that there were unnanotated exons and 3′ untranslated regions (UTR) of the *TRPM2* gene expressed in leukocytes (Fig. 1a). This observation promoted us to ask whether the *TRPM2* isoforms were differentially expressed between horses with different *TRPM2* genotypes as described above. To test this, we first merged the transcriptomes of each animal to increase the total depth of coverage of the transcriptomes and used *Cufflinks* to assemble transcripts of *TRPM2*. We then examined differential expression of the assembled *TRPM2* transcripts as described above. The transcript assembly revealed 2 unspliced 5′ and 3′ transcripts of *TRPM2* (hereafter referred to as *TRPM2-1* and *TRPM2-2)* that included 17 novel isoforms (*TRPM2-1* = 10, *TRPM2-2* = 7) consisting of unnanotated exons (e.g., 5′ terminal and cassette exons), retained introns, and 3′ UTRs (Fig. 1b). Neither *TRPM2-1* nor *TRPM2-2* were differentially expressed between the 2 cohorts (*edgeR* and *cuffdiff*). Likewise, no differentially expressed isoforms were identified between the 2 groups. We then examined differential expression of the *TRPM2* transcripts among the 3 *TRPM2* genotypes (AA vs AB; AA vs BB; AB vs BB). The *TRPM2-1* transcript was significantly higher in the AB group in comparison to the AA group as well as higher than AA in all comparisons (Table 1).

**Table 1:** Pairwise comparisons of potentially novel *TRPM2* transcript expression values

Cufflinks ID	Transcript ID	AA	AB/BB	Log_2_ FC	*P* value	P adjusted
CUFF.23429	*TRPM2-1*	90.99	118.90	0.39	0.0971	0.1942
CUFF.23430	*TRPM2-2*	168.23	214.17	0.35	0.1630	0.3260
		AA	AB			
CUFF.23429	*TRPM2-1*	91.81	130.55	0.51	0.0224	0.0448
CUFF.23430	*TRPM2-2*	169.73	225.94	0.41	0.0783	0.1566
		AA	BB			
CUFF.23429	*TRPM2-1*	90.63	110.04	0.28	0.2663	0.5326
CUFF.23430	*TRPM2-2*	167.57	205.44	0.29	0.2766	0.5532
		AB	BB			
CUFF.23429	*TRPM2-1*	128.83	110.27	−0.22	0.3456	0.6912
CUFF.23430	*TRPM2-2*	223.02	205.87	−0.12	0.6565	1.0000

To determine whether the 2 predicted genes at the annotated at *TRPM2* locus are in fact 2 independent transcriptional units, we isolated RNA, synthesized cDNA, and probed with primers targeted for the 3′ end of *TRPM2-1* and the 5′ end of *TRPM-2*. Gel electrophoresis of the amplicons revealed that PCR successfully amplified transcripts spanning the region, indicating that *TRPM2-*1 and −2 are, at least in some instances, spliced together as a single transcription unit (Fig. 1c). Visual inspection of the PCR gel revealed several splice variants (amplicons) synthesized between the primer sets which corresponds with the several exons predicted by the Ensembl gene prediction track. This analysis does not rule out the possibility that independent transcriptional units might be synthesized from the *TRPM2-1* and −*2* locus.

### Differential gene expression analysis of transcriptome (untargeted approach)

**Fig. 2:**
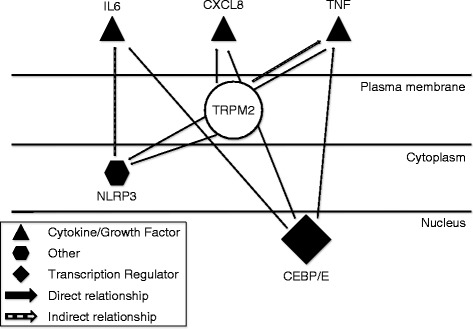
*EdgeR* pathway analysis results. *TRPM2* was not identified as differentially expressed in the analysis but was added for the purpose of identifying *TRPM2’s* link with the differentially expressed genes. Solid arrows indicate a documented direct interaction, while dashed arrows represent interactions linked through an intermediary. The transcription regulator CEBP/E directly interacts with IL6, CXCL8, and TNF establishing a link between the homozygous AA *TRPM2* horses and expression of immune related genes. The indirect interactions show the relationship of *TRPM2* to the differentially expressed genes and thus suggesting a role for TRPM2 in innate immunity. Figure adapted from IPA generated output for resolution purposes

Examination of the gene count tables generated by *HTSeq* revealed 8212 genes with < 1 read count/gene, leaving 18,779 genes for analysis in *edgeR*. Comparison of the *TRPM2* genotypes (AA [*n* = 3] vs. AB [*n* = 4] + BB [*n* = 5]) showed that expression of the ankyrin repeat domain 22 (*ANKRD22*)*,* major histocompatibility complex, class II, DQ beta (*DQB*)*,* and myeloperoxidase (*MPO*) genes were significantly higher (FDR ≤ 0.05) in horses with the *TRPM2* AA genotype compared to horses with the *TRPRM2* AB and BB genotypes (Table 2). *Cuffdiff* analysis revealed 58 differentially expressed genes between the 2 groups (Table 3); however, none of the genes were concordant between the 2 analyses. Although not significantly different, expression of *DQB* was closest to being differentially expressed (*P* = 0.00075, FDR = 0.108) in the *cuffdiff* analysis, with the *ANKRD22* and *MPO* having FDRs of 0.73 and 0.63, respectively. Pathway analysis of the differentially expressed genes identified by *cuffdiff* revealed biological processes involving host antimicrobial and inflammatory response (17 genes), cell-to-cell signaling, and cell interaction (Fig. 2). Notably, the CCAAT/enhancer binding protein epsilon (*C/EBPE*), which was a central gene in the identified pathways, was expressed ~ 1.3-fold higher in the horses with *TRPM2* AA genotypes relative to the other horses. No biological processes were identified using the differentially expressed genes identified by *edgeR*. Comparison of transcriptome by clinical status as foals (i.e., *R. equi* pneumonia [*n* = 2], unaffected [*n* = 10]) using the analysis methods described above identified no differentially expressed genes (FDR ≤ 0.05).

**Table 2:** Transcriptome-wide differentially expressed genes identified by *edgeR* analysis

Ensembl ID	Gene name	Log FC^a^	Log CPM^b^	*P* value	FDR
ENSECAG00000022239	ANKRD22	−5.82	0.23	0.0000	0.0000
ENSECAG00000006492	DQB	−2.00	7.30	0.0000	0.0028
ENSECAG00000006662	MPO	−3.22	−0.44	0.0000	0.0483
ENSECAG00000017147	C15orf52	−3.08	−1.66	0.0000	0.0968
ENSECAG00000002249	PLEKHG4B	−4.64	−1.57	0.0000	0.0983
ENSECAG00000008171	N/A	−3.80	−0.68	0.0000	0.1476
ENSECAG00000008238	S100A5	−3.41	−1.98	0.0001	0.1476
ENSECAG00000016666	OMG	0.89	6.37	0.0001	0.2650
ENSECAG00000006656	N/A	−3.08	−2.89	0.0002	0.4258
ENSECAG00000024043	CSTA	−2.09	2.50	0.0003	0.4904

^a^ *FC* fold change

^b^ *CPM* counts per million

**Table 3:** Transcriptome-wide differentially expressed genes identified by *cuffdiff* analysis

Ensembl ID	Gene name	Log_2_ FC	*P* value	Q value	Significant
ENSECAG00000024043	CSTA	−2.08	0.0001	0.0122	yes
ENSECAG00000024259	DQA	−1.83	0.0001	0.0122	yes
ENSECAG00000019922	ADAMDEC1	−1.39	0.0001	0.0122	yes
ENSECAG00000009142	DQA	−1.34	0.0001	0.0122	yes
ENSECAG00000020816	PDLIM1	−1.32	0.0001	0.0122	yes
ENSECAG00000015109	N/A	−1.28	0.0001	0.0122	yes
ENSECAG00000012883	CEBPE	−1.27	0.0001	0.0122	yes
ENSECAG00000023062	N/A	−1.26	0.0001	0.0122	yes
ENSECAG00000019130	SIRPG	−1.22	0.0001	0.0122	yes
ENSECAG00000011315	EMR3	−1.17	0.0001	0.0122	yes
ENSECAG00000013660	C1orf186	−1.16	0.0002	0.0371	yes
ENSECAG00000025078	SUSD2	−1.05	0.0001	0.0122	yes
ENSECAG00000016730	CSMD1	−0.99	0.0001	0.0122	yes
ENSECAG00000001282	CCR3	−0.95	0.0001	0.0122	yes
ENSECAG00000008322	GZMH	0.79	0.0002	0.0371	yes
ENSECAG00000007621	TRIP11	0.86	0.0003	0.0435	yes
ENSECAG00000018564	SPATS2L	0.87	0.0001	0.0221	yes
ENSECAG00000019111	CD163	0.89	0.0002	0.0308	yes
ENSECAG00000020763	MGST1	0.91	0.0001	0.0221	yes
ENSECAG00000014422	OAS2	0.92	0.0001	0.0122	yes
ENSECAG00000011776	MX1	0.93	0.0001	0.0221	yes
ENSECAG00000021989	DDX58	0.94	0.0002	0.0308	yes
ENSECAG00000003474	TTLL3	0.94	0.0001	0.0122	yes
ENSECAG00000001399	SAMD9	1.03	0.0001	0.0221	yes
ENSECAG00000014218	SIGLEC1	1.04	0.0003	0.0435	yes
ENSECAG00000008274	CLEC4E	1.05	0.0001	0.0122	yes
ENSECAG00000010117	S100A9	1.05	0.0001	0.0122	yes
ENSECAG00000013762	NCR1	1.07	0.0002	0.0308	yes
ENSECAG00000023733	MMP-1	1.10	0.0001	0.0221	yes
ENSECAG00000015006	FGFR1	1.10	0.0003	0.0435	yes
ENSECAG00000007133	TPPP3	1.11	0.0001	0.0122	yes
ENSECAG00000022042	PNP	1.11	0.0001	0.0122	yes
ENSECAG00000008356	ZNF577	1.15	0.0003	0.0435	yes
ENSECAG00000012235	BAZ2B	1.15	0.0001	0.0122	yes
ENSECAG00000009742	S100A12	1.15	0.0001	0.0122	yes
ENSECAG00000021476	MMP8	1.16	0.0001	0.0122	yes
ENSECAG00000009271	S100A8	1.18	0.0001	0.0122	yes
ENSECAG00000019411	HERC6	1.20	0.0001	0.0122	yes
ENSECAG00000026820	SEPP1	1.22	0.0001	0.0122	yes
ENSECAG00000007881	IFIH1	1.24	0.0001	0.0122	yes
ENSECAG00000014645	OASL	1.29	0.0001	0.0122	yes
ENSECAG00000010153	IFIT4	1.33	0.0001	0.0122	yes
ENSECAG00000013874	SPARC	1.44	0.0002	0.0308	yes
ENSECAG00000001481	SAMD9L	1.47	0.0001	0.0122	yes
ENSECAG00000008809	OAS3	1.51	0.0001	0.0122	yes
ENSECAG00000000500	IF16	1.53	0.0001	0.0122	yes
ENSECAG00000015395	HERC5	1.54	0.0001	0.0122	yes
ENSECAG00000001324	ISG15	1.55	0.0001	0.0122	yes
ENSECAG00000008594	BTN3A1	1.59	0.0001	0.0122	yes
ENSECAG00000006913	N/A	1.90	0.0001	0.0122	yes
ENSECAG00000019949	CYP4F	1.92	0.0001	0.0122	yes
ENSECAG00000017437	MYLPF	2.38	0.0002	0.0371	yes
ENSECAG00000004349	IFIT5	2.54	0.0001	0.0122	yes
ENSECAG00000020407	MYBPC2	2.64	0.0002	0.0371	yes
ENSECAG00000010020	HBB	2.73	0.0001	0.0122	yes
ENSECAG00000023971	TNNT3	4.46	0.0002	0.0371	yes
ENSECAG00000019728	TNNC2	5.11	0.0001	0.0122	yes
ENSECAG00000005487	N/A	8.08	0.0001	0.0122	yes

## Discussion

As previously reported, *TRPM2* markers have been associated with clinical disease caused by *R. equi* [15]. The *TRPM2* gene was biologically plausible as a candidate gene because TRPM2 has been shown to increase tissue damage at sites of inflammation in a mouse model of ulcerative colitis [15, 31]. On the basis of these findings, we considered it important to understand the expression pattern across the region of interest identified by our previous GWAS (targeted approach). We found evidence indicating that alternative splicing occurs within the *TRPM2* locus in horses resulting in multiple isoforms. Although we did not attempt to verify the functionality of these transcripts in horses, an alternative transcript has been functionally characterized in human-derived cell lines: a short isoform of *TRPM2* inhibits calcium influx while increasing cell viability [32]. Given our demonstration of only a single *TRPM2* gene in the equine genome, it is biologically plausible that the short isoforms of *TRPM2* in the horse might have altered function that could confer some degree of protection against infection and subsequent tissue damage. Further experimentation is required to understand the function of these isoforms of equine *TRPM2* and what role these isoforms might play in innate immune responses to *R. equi* infection.

The *C/EBP* gene was identified as differentially expressed by *cuffdiff* (Table 3) and was indirectly implicated by *IPA* (Fig. 2). Like *TRPM2*, this gene is associated with neutrophil function. Isoforms of the *C/EBP* gene are expressed by human neutrophils and play an integral part in inducing several inflammatory cytokines [33]. C/EBP was shown to bind the promoter of interleukin 8 (*IL-8, CXCL8*) when stimulated by lipopolysaccharides (LPS), [33] and upregulation of *CXCL8* production by TRPM2 has been linked to increased tissue damage at sites of inflammation in mice [31, 34].

The transcriptome-wide analysis for association of gene expression with *TRPM2* SNP genotype using both *edgeR* and *cuffdiff* did not reveal any concordant results. This might be attributable to several factors. Each program uses a different method to predict differentially expressed genes and each is tailored to conduct different types of analysis. *Cuffdiff* uses a mixture of distributions to account for the uncertainty in mapping a read and the variability in read count while *edgeR* primarily focuses on the variability in read count across replicates [21, 22]. The results from *edgeR* appear to be more conservative in our experiment because only 3 genes were identified as differentially expressed whereas *cuffdiff* identified 58 genes. There is also evidence to suggest that *edgeR* is not always a more conservative approach; thus, the observed discrepancy might reflect some other biological or technical property present in the data but no other metrics suggest this was the case [35]. Also, *cuffdiff* uses the alignments from *TopHat* and a gene annotation file to calculate differential expression. This removes the *HTSeq* count step and might have contributed to the differing results. In an effort to maximize the inferences drawn from our data, we elected to use the genes identified by *cuffdiff* to better leverage our data in elucidating biological pathways and processes playing a role in susceptibility to *R. equi* pneumonia.

There are several other factors to consider regarding RNA-Seq and the outcome of its analysis. RNA-Seq reflects steady-state levels of RNA, which encompasses the rate of transcription, rate of degradation, post-transcriptional phases, and post-translational modifications that may alter protein function [36]. For example, a 1.3-fold increase in expression of *C/EBP* within the AA genotypes group over the non-AA genotypes is a complex finding, as it is in any case investigating differentially expressed genes. A first step in following up on the findings and towards a better understanding this gene and pathway as it relates to *R. equi* pneumonia would be to confirm the RNA expressions levels by another method. Also, we do not know whether a 1.3-fold increase in RNA represents a 1.3-fold increase in the protein level of C/EBP in these horses. A recent study found that on average only 40% of the variability in protein levels of the cell could be explained by mRNA levels [37]. This finding demonstrates that the dynamic processes of transcription and translation cannot be easily generalized to their functional implications.

Our transcriptome-wide analysis of gene expression with disease status compared the samples based on a horse’s phenotypic classification when it was a foal. No evidence that disease status as a foal was associated with gene expression as an adult; however, our study was clearly lacking power and results of absence of evidence of an association should not be confused with evidence of absence of an association.

Our study had a number of limitations. This project was conducted using mature horses and not foals: gene expression as adults might not reflect the gene expression of these horses when they were foals and susceptible to *R. equi*. The marker genotypes (*i.e.*, AA, AB, and BB), however, would not have changed and therefore their impact on functional transcription should remain the same. A second limitation is that the region of interest’s involvement with and relationship to *R. equi* infection might be tissue-specific (*e.g.*, in the lung or in alveolar macrophages) and thus not captured or accurately reflected by RNA-Seq data from whole blood. A third limitation is the small sample size of the study which was dictated by available funding. This was particularly true for our secondary aim comparing gene expression by pneumonia status as a foal. Nevertheless, previous studies have yielded important results using fewer horses than this and our results provide novel, significant findings relevant to understanding the pathogenesis and susceptibility of foals to *R. equi* infection [18–20].

To the authors’ knowledge, these data represent the first whole transcriptome assembly of the Quarter Horse genome. With an average of 33.7 million reads per sample, we have generated an average of 53.83X coverage of the Quarter Horse transcriptome. The *cufflinks* program estimated over 34,000 genes to be present in the Quarter Horse genome. Furthermore, *cufflinks* estimated over 74,500 isoforms of these 34,000 genes to be transcribed. The RNA-Seq data presented in this report should be valuable to those interested in the equine transcriptome because researchers will be able to align their transcriptomic/genomic data with the Quarter Horse transcriptome to further investigate functional implications of comparative genomics.

## Conclusions

*Rhodococcus equi* is a pathogen that predominantly affects young foals with often severe and potentially fatal outcomes. Here we described several differentially expressed genes, identified using RNA-Seq, associated with this genotype that are important to innate immune responses. Specifically, *C/EBP* was found to be upregulated in horses with the susceptible genotype. This is an important finding due to the role *C/EBP* plays in enhancing *IL-8* expression as increased concentration of *IL-8* at sites of inflammation has been shown to increase tissue damage. These findings suggest that modulation of expression of *TRPM2* contributes to susceptibility to R. equi pneumonia and has shed further light on our understanding of the susceptibility genotype and its relationship to gene expression.

## Abbreviations

*ANKRD22*: Ankyrin repeat domain 22
Bp: Base-pair
*C/EBPE*: CCAAT/enhancer binding protein epsilon
*DQB*: Major histocompatibility complex, class II, DQ beta
FDR: False discovery rate
Fe^2+^: Iron
GWAS: Genome-wide association study
*IL-8, CXCL8*: Interleukin 8
IPA: Ingenuity Pathway Analysis
LPS: Lipopolysaccharide
*MPO*: Myeloperoxidase
PCR: Polymerase chain reaction
PE: Paired-end
*R. equi*: *Rhodococcus equi*
RNA-Seq: RNA-Sequencing
SNP: Single nucleotide polymorphism
*TRPM2*: Transient receptor potential cation channel, subfamily M, member 2
UCSC: University of California, Santa Cruz
UTR: Untranslated region
VapA: Virulence associated protein
